# Sulforaphane: An emergent anti-cancer stem cell agent

**DOI:** 10.3389/fonc.2023.1089115

**Published:** 2023-01-23

**Authors:** Leandro de Lima Coutinho, Tharcísio Citrangulo Tortelli Junior, Maria Cristina Rangel

**Affiliations:** Centro de Investigação Translacional em Oncologia (LIM24), Departamento de Radiologia e Oncologia, Instituto do Câncer do Estado de São Paulo, Faculdade de Medicina da Universidade de São Paulo, São Paulo, SP, Brazil

**Keywords:** natural products, conventional therapy, cancer stem cells, chemoprevention, anticancer

## Abstract

Cancer is a major public health concern worldwide responsible for high morbidity and mortality rates. Alternative therapies have been extensively investigated, and plant-derived compounds have caught the attention of the scientific community due to their chemopreventive and anticancer effects. Sulforaphane (SFN) is one of these naturally occurring agents, and studies have shown that it is able to target a specific cancer cell population displaying stem-like properties, known as cancer stem cells (CSCs). These cells can self-renewal and differentiate to form highly heterogeneous tumor masses. Notably, most of the conventional chemotherapeutic agents cannot target CSCs once they usually exist in a quiescent state and overall, the available cytotoxic drugs focus on highly dividing cells. This is, at least in part, one of the reasons why some oncologic patients relapse after standard therapy. In this review we bring together studies supporting not only the chemopreventive and anticancer properties of SFN, but especially the emerging anti-CSCs effects of this natural product and its potential to be used with conventional antineoplastic drugs in the clinical setting.

## 1 Introduction

Cancer is one of the most life-threatening conditions that affect human health. According to GLOBOCAN, it is estimated that the global cancer burden will be 28.4 million cases in 2040, which means a 47% increase in comparison to 2020 rates, with a higher growth in transitioning countries (64% to 95%) (1).

Even though oncologic therapy has improved throughout the last decades, the development of drug resistance, tumor relapse and metastasis are still major concerns of conventional therapies (2). One of the reasons for this is the existence of a subpopulation of cancer cells that display unique features such as self-renewal and differentiation capabilities. These cells are known as Cancer Stem Cells (CSCs) or Tumor Initiating Cells (TICs) and are able to hierarchically originate differentiated cancer cells and recapitulate the heterogeneity of the primary tumor (3). Once CSCs can enter a quiescent state acquiring a protective form, most of the available anticancer therapies available in the clinic cannot effectively target this subpopulation of cancer cells. Therefore, after standard therapy, CSCs may remain in the tumor bed and eventually lead to relapse (4).

Considering that CSCs may ultimately promote drug resistance and tumor relapse, alternative chemopreventive and therapeutic approaches are needed. In line with this, plant-derived compounds have caught the attention of the biomedical community (5). Also, the combination of natural products with conventional drugs used in cancer treatment have been widely studied over the last decades aiming to optimize or improve therapeutic responses to different chemotherapeutics agents (6).

**Figure 1:**
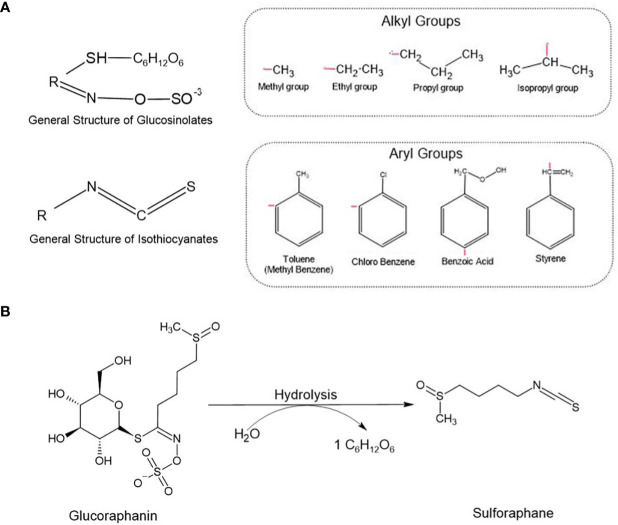
**(A)** Glucosinolates and Isothiocyanates general structures. The radical ‘R’ represents different alkyl and aryl groups. **(B)** Glucoraphanin, a glucosinolate, is converted into sulforaphane (SFN) by the enzyme myrosinase. During the chemical reaction, glucoraphanin is hydrolyzed releasing a glucose molecule (C_6_H_12_O_6_) and resulting in the active form of SFN.

Here we review studies supporting the chemopreventive and anticancer effects of SFN, with special emphasis on its anti-CSC properties. We discuss the ability of SFN to modulate the self-renewal of CSCs and to influence some signaling pathways aberrantly activated in these cells (**Figure 1**). Finally, we present evidence supporting the use of SFN as an alternative and/or complementary therapeutic agent in a variety of cancers.

## 2 Isothiocyanates and cancer

Isothiocyanates (ITCs) are biologically active small molecules derived from glucosinolates (GSLs). There are hundreds of GSLs in cruciferous plants, thus resulting in the yield of several ITCs as their secondary metabolites. The general chemical structure of an ITC is R–N=C=S, where R stands for an alkyl or aryl group (**Figure 1A**). Cruciferous vegetables, such as cauliflower, cabbage, and broccoli, are the main source of these secondary metabolites capable of mediating different biological processes including oxidative stress and detoxification. Many of these compounds display anticancer properties, and studies have been conducted to investigate their preventive and therapeutic potentials against a variety of cancers (7–9).

Sulforaphane (SFN) is an ITC found in its inactive storage form as glucoraphanin, and its major source is broccoli, an important plant from the family *Brassicaceae*. Upon mechanical damage (e.g., biting, chewing, or slicing) of broccoli and other cruciferous vegetables, glucoraphanin is released and hydrolyzed by the plant enzyme myrosinase, forming its active molecule SFN (**Figure 1B**). When exposed to high temperatures during meal preparation, myrosinase can be degraded, lose its function, and subsequently compromise the synthesis of SFN. Thus, eating raw cruciferous vegetables, instead of heating them can significantly improve the biodisponibility of SFN and its subsequent beneficial effects. Also, when consuming the natural extracts, it is important to choose those presenting myrosinase-activated glucoraphanin to guarantee SFN biosynthesis. Although some studies have shown that myrosinase can be found in bacteria from the gut microbiota and that these microorganisms can hydrolyze GLSs into their active forms, this conversion is not so efficient as the one mediated by the plant-derived myrosinase (10).

Over the last few years, SFN has been intensely investigated for its therapeutic potential. It has been suggested that, compared to other vegetables and fruits, cruciferous vegetables are efficient in preventing cancer development. In addition, epidemiological studies have shown that SFN exerts chemopreventive and anticancer properties against different tumors (11).

## 3 Chemopreventive properties of sulforaphane

*In vitro* and *in vivo* studies have reported that SFN exhibits chemopreventive and anticancer properties against different cancer types including those of the lung, prostate, colon, and breast (12). The use of plant-derived products for treating cancer is termed “green chemoprevention”. These compounds can prevent, block, or even revert tumor initiation processes before clinical manifestation (11). On the other hand, anticancer agents, as the name implies, refer to any drug or compound that is able to treat malignant or cancerous diseases once they are clinically manifested (13).

In a study aiming to assess SFN chemopreventive properties against colon cancer, it was reported in a mouse model of azoxymethane-induced colon tumors that daily treatment with SFN for 8 or 24 weeks exhibited a significant reduction in aberrant crypt foci (ACF) formation. In addition, daily intake of SFN for 24 weeks was able to decrease the number of colonic ACF in patients with colonic adenoma. SFN effects on patients’ gut microbiota was also reported in the same study, where the intake of broccoli sprouts (BS) for 2 weeks was able to increase the population of two beneficial bacteria, *Bifidobacterium* and C*lostridium cluster VIa*. Both species benefit human health through effects including the protection of colonic mucosa by enhancing the synthesis of butyrate (14).

Moreover, the chemopreventive effects of SFN were also reported by Castro et al. (15) in a Triple-negative Breast Cancer (TNBC) mouse model. The authors studied Balb/C nude mice engrafted with MDA-MB-231-Luc-D3H1 cells and categorized them into two treatment groups: pre- and post-treated with SFN. The pre-treated group received daily intraperitoneal injections of SFN (50 mg/Kg) for 2 weeks prior tumor cells inoculation, and the treatment was kept for 3 more weeks. The post-treatment group was treated with SFN for 3 weeks after tumor cells inoculation. Tumor volume was reduced by 29% in the SFN pre-treated group as compared to saline-treated controls, while there was only a 14%-tumor reduction in the SFN post-treated group. These findings highlight the chemopreventive properties of SFN, once its administration to mice prior tumor challenge results in a higher rate of tumor growth inhibition (15).

There are several mechanisms underlying the chemopreventive effects of SFN, including the induction of Phase II enzymes [glutathione S-transferase (GST), N-acetyltransferase (NAT), and sulfotransferase (SULT)] detoxification, and Phase I enzymes (cytochrome P450, CYP) inhibition, frequently involved in carcinogenic activation (16). Consequently, SFN can prevent DNA adducts formation, decreasing the accumulation of DNA damage and possible mutations. This herb-derived agent can also promote cell cycle arrest and apoptosis by regulating different signaling pathways including Nuclear Factor erythroid Related Factor 2 (Nrf2)-Keap1 and NF-κB. In addition, recent findings show that SFN is able to modulate the activity of some epigenetic factors, such as histone deacetylases (HDAC), thus impacting the expression of genes involved in tumor initiation and progression (17).

## 4 Anticancer effects of sulforaphane

Besides the chemoprevention properties of SFN, over the last decades, its anticancer effects have been widely reported by different *in vitro* and *in vivo* studies against different cancer types including breast, ovarian, prostate, colon, lung, and gastric cancer (15, 18–21).

SFN was able to decrease the proliferation and invasion of MDA-MB-231 TNBC cells co-cultured with tumor-associated macrophages (TAMs) by disrupting the communication between these two cell types (22). Importantly, cancer cells were able to recruit TAMs to compose their tumor microenvironment (TME) by secreting signal molecules (e.g., CSF-2) and interleukins (e.g., IL-6, IL-8, IL-10, etc.). Once in the TME, TAMs supported cancer development through a variety of signaling pathways such as TGF-β, fibroblast growth factor 2 (FGF2), and vascular endothelial growth factor (VEGF) (23).

In addition, SFN induced cell cycle arrest, apoptosis, upregulation of p21 and p27, and promoted senescence in the breast cancer cells MCF-7, MDA-MB-231, and SK-BR-3 by inducing global DNA hypomethylation and inhibiting the DNA methyltransferases DNMT1 and DNMT3B (24). Moreover, SFN was able to decrease the expression of *DNMT1* and *DNMT3* in LnCap prostate cancer cells, as well as to reduce methylation in *Cyclin D2* promoter, thus inducing *Cyclin D2* gene expression in those cells. Cyclin D2 is a regulatory factor of the cell cycle and hypermethylation of its promoter is associated with prostate cancer progression. Restoration of *Cyclin D2 expression by* SFN resulted in antiproliferative effects in LnCap cells (25).

SFN anticancer effects were also reported in colon cancer cells. In HCT-116 colon cancer cells lacking p53, HCT-116 p53KO, SFN induced DNA damage, enhanced Bax expression and the release of cytochrome C followed by apoptosis (26). In addition, SFN increased reactive oxygen species (ROS), apoptosis-inducing factor (AIF), and promoted cell cycle arrest at the G2/M phase by reducing CDK1 protein levels. SFN also exerted effects on caspase activity, including the activation of caspase-3, -8, and -9 (27). All these results highlight the pro-apoptotic effects of SFN and its potential to repress colon cancer progression.

In lung cancer, SFN was found to increase the apoptotic and necrotic populations of lung cancer cells in a dose-dependent manner, and induced cell cycle arrest at the S phase. Lung cancer cells treated with SFN displayed higher *Cyclin D1* and *Cyclin K* expression. Both genes are involved in cell cycle progression and their expression positively correlates with cell proliferation. However, when assessing the prognostic impacts of *Cyclin K* overexpression in patients with lung adenocarcinoma, there was no association with overall survival (OS) as exhibited by Kaplan-Meier plotter curves (28, 29). The effects of SFN in non-small cell lung cancer (NSCLC) seem to be related to the epidermal growth factor receptor (EGFR) status. NSCLC cells expressing high (CL1-5 cells) and low (CL1-0 cells) levels of EGFR responded differently to SFN. CL1-5 cells are resistant to SFN treatment, while the low levels of EGFR in CL1-0 were associated with an increase in SFN-induced ROS levels followed by cell apoptosis (30). Additionally, SFN metabolites were able to inhibit cell migration and invasion by modulating different CLAUDIN isotypes and promoting microtubule disruption in lung cancer cells (SK-1 and A549) (19).

Additionally, SFN was found to decrease sarcoma-associated herpesvirus (KSHV)-infected primary effusion lymphoma (PEL) cells by possibly inhibiting the phosphorylation of p38 mitogen-activated protein kinase (p38MAPK) and AKT, which are crucial pro-oncogenic pathways involved in cell proliferation, migration, invasion, and cell survival (31). SFN also showed antitumor effects in a chemical-induced skin carcinogenesis model, by blocking sulfatase-2 activity and subsequently increasing heparan sulphate proteoglycans (HSPGs) and reducing glypican-3. HSPGs are components of the extracellular matrix (ECM) that promote cell-cell or cell-ECM interactions, thus inhibiting cancer migration and invasion. Moreover, SFN significantly activated the major antioxidant marker Nrf2 and decreased NFκB, TNF-α, IL-1β and caspase-3 at transcriptional and protein levels (32).

## 5 Cancer stem cells and sulforaphane

Besides the studies supporting the notion that SFN can inhibit tumor development and/or diminish cancer progression through different mechanisms, there is growing evidence that SFN can target CSCs of different tumors. CSCs are believed to be responsible for the initiation, promotion, progression, and the maintenance of tumors, thus considered the ‘fuel’ of carcinogenesis (33).

CSCs exhibit a stemness phenotype characterized by self-renewal and differentiation capacities which give these cells the ability to recapitulate the complex heterogeneity of primary tumors. In addition, CSCs are intrinsically resistant to conventional therapies including chemo and radiotherapy, that mainly target high proliferative cells. CSCs are usually in a low-cycling quiescent state, which makes them almost unaffected by conventional cytotoxic agents (34).

Additionally, CSCs plasticity is one of the major challenges in the treatment of cancer, since those cells can transit between a poorly- to a well-differentiated state and vice-versa (35). Furthermore, CSCs are involved in cell migration, invasion, metastasis, angiogenesis, and tumor relapse, thus greatly impacting patient outcomes (36, 37). Therefore, targeting CSCs may represent a promising strategy in cancer treatment by preventing therapy resistance, metastasis, and tumor relapse.

**Figure 2:**
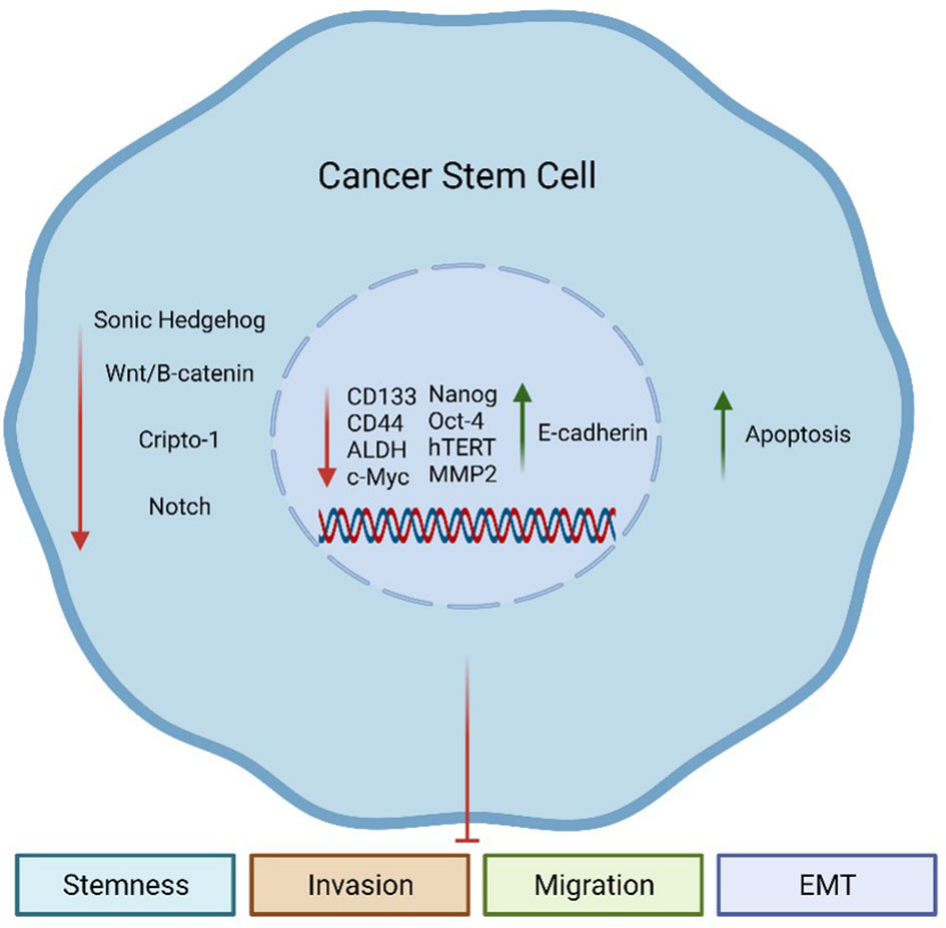
Sulforaphane (SFN) effects on Cancer Stem Cells (CSCs). SFN can inhibit aberrantly activated embryonic pathways in CSCs, including Sonic Hedgehog (SHH), Wnt/β-catenin, Cripto-1 (CR-1), and Notch. Additionally, SFN can decrease the expression of CSC-related genes such as *CD133*, *CD44*, *ALDH*, *c-Myc*, *Nanog*, *Oct-4*, *hTERT*, and *MMP2*. These genes are involved in the maintenance of the CSC population. On the other hand, SFN can increase the expression of E-cadherin, an epithelial marker. All these events together culminate in an increase of apoptosis, while reducing the stemness phenotype of CSCs and their capacity to invade, migrate, and undergo the epithelial-to-mesenchymal (EMT) process.

Remarkably, herb-derived agents, such as SFN, have attracted attention due to their anti-CSC effects (**Figure 2**) in different types of cancer as discussed in the following topics.

### 5.1 Leukemia

CSCs were first reported in liquid tumors, such as leukemias, with CD34^+^/CD38^−^ cancer cells capable of initiating and promoting tumorigenesis in immunodeficient mice injected with cells derived from Acute Myeloid Leukemia (AML) patients (38). Later, another group showed evidence that AML would follow a hierarchical development pattern with CSCs on the top and giving rise to differentiated cancer cells by asymmetric division, originating the heterogeneity observed in AML patients (39). Notably, CSCs have been associated with therapy resistance of AML to standard therapy through a variety of mechanisms, such as activation of embryonic pathways including Sonic Hedgehog (SHH) and Wnt/β-catenin, leading to cell dormancy and apoptosis resistance (40). SFN was found to inhibit the proliferation of AML stem-like cells *in vitro* and *in vivo*, by possibly decreasing the transcriptional and protein levels of components of the SHH pathway, one of the embryonic signaling pathways found to be dysregulated in CSCs. By impairing SHH, SFN decreased the self-renewal of AML stem-like cells, thus affecting their proliferation (41).

### 5.2 Lung cancer

The basis for studying CSCs in lung cancer comes from a study of 1981, in which the authors reported that 0.05 to 1.5% of cells derived from patients with adenocarcinoma and small cell carcinoma of the lung exhibited colony formation capacity *in vitro* and were able to form and recapitulate the heterogeneity of the primary tumor *in vivo* when transplanted into nude mice (42).

Extensive research has been made in order to target CSCs in lung cancer and improve response to therapy (43). Experimental models have pointed SFN as a promising herb-derived anti-CSC agent in this type of cancer. SFN inhibited self-renewal and proliferation of CD133^+^ cells derived from the lung cancer cell lines A549 and H460. mRNA and protein levels of SHH and its downstream targets Smo and Gli1, as well as polyhomeotic homolog 3 (PHC3), were highly elevated in the CD133^+^ population. SHH pathway is one of the embryonic signaling pathways abnormally activated in CSCs that may crosstalk with PHC3 promoting self-renewal of lung CSCs. However, upon SFN treatment SHH, Smo, Gli1, and PHC3 activities were reduced, thus impacting the maintenance of the stemness phenotype of lung CSCs (44).

SFN was reported to inhibit EMT and metastasis in the NSCLC, the cell lines H1299, 95C, and 95D, by decreasing the expression of *miR-616-5p.* This *miR* directly targets *GSK3β* and decreases its expression resulting in the accumulation of β-catenin in the cytoplasm, which in turn activates Wnt signaling pathway, thus promoting CSCs survival. SFN was able to indirectly inhibit Wnt/β-catenin pathway by reducing the expression of *miR-616-5p*. In addition, SFN decreased proliferation of all NSCLC cells, and reduced migration and invasion of 95D and H1299 cells at low doses (1–5 μmol/L) (45).

c-Myc plays an important role in regulating lung cancer cell growth, death, and treatment resistance, and also promotes cell migration, invasion, EMT, and the activation of Notch and Wnt/β-catenin pathways, thus directly impacting the maintenance of CSCs (46). SFN was shown to inhibit CSC-like properties and improve the efficacy of CIS treatment in the NSCLC cells H460, H1299, and A549. Mechanistically, SFN induced the expression of *miR-214*, which binds to the coding region of *c-Myc* and represses it. Additionally, SFN reversed CIS-induced endogenous c-Myc accumulation, which is associated with enhanced CIS cytotoxicity in NSCLC cells either *in vitro* or *in vivo*. These findings suggest that SFN may be potentially used as a co-adjuvant therapy along with CIS in lung cancer patients or in any c-Myc-addicted tumor (47).

### 5.3 Breast cancer

Breast cancer was the first solid tumor in which CSCs were indeed reported (48). In this study, the authors identified a subpopulation of cancer cells characterized by the ability to initiate tumors. Only those cells expressing the immunophenotype CD44^+^/CD24^-^ were able to form tumors when transplanted into immunocompromised mice. As few as 100 cells expressing the immunophenotype CD44^+^/CD24^-^ were able to form tumors when injected in those mice, while thousands of cells with alternative phenotypes did not succeed (48). This pioneering work made room for the development of different studies in order to better understand the roles of CSCs in breast cancer initiation and progression and also raised the need to search for new treatment approaches targeting this unique cell population.

SFN was found to inhibit proliferation and mammosphere formation in TNBC cells and decreased the expression of the pluripotent marker Cripto-1 (CR-1) in these cells (15). *CR-1 i*s an embryonic gene aberrantly expressed during carcinogenesis and promotes the acquisition of stemness traits by inducing *Nanog*, acetaldehyde dehydrogenase (*ALDH1A1*), *Wnt3*, and *Notch4*, other CSC-related genes inhibited by SFN treatment (15). SFN was able to control breast cancer progression by modulating CR-1 signaling pathway in tumor-bearing mice, and also inhibited CR-1 binding to Activin receptor type-IB (ALK4), thus blocking its downstream signal transduction (15).

Epithelial-to-mesenchymal transition (EMT) is defined as a reversible process in which cells lose their polarity and cell-cell adhesion, gain migratory capacity, and acquire mesenchymal, fibroblast-like properties. These biological events are crucial for cancer cells to metastasize surrounding tissues or distant organs (49). EMT is a key process during cancer progression and metastasis and seems to be related to CSCs (35, 50). MDA-MB-231 cells represent the mesenchymal subtype of TNBC and is characterized by an aggressive, poorly differentiated, and highly invasive phenotype associated with the EMT process (51). Aiming to assess the effects of SFN in MDA-MB-231 cells, Bagheri et al. (52) reported that SFN inhibited cells migration starting at the lower concentration of 5 μM, while induced cell apoptosis only at relatively high doses (30 and 40 µM). Additionally, cells treated with SFN exhibited reduced expression of the EMT markers *Fibronectin* (starting at 20 µM) and *ZEB1* (40 µM), and decreased protein accumulation of *β-catenin* in a time-dependent fashion upon 40 µM SFN treatment (52).

Importantly, breast CSCs can transit towards two distinct states: CD44^+^/CD24^-^ quiescent mesenchymal-like and ALDH^+^ proliferative epithelial-like states (53). SFN has been found to decrease the ALDH^+^ breast cancer cell population by 65~80% and reduce the size and number of primary mammospheres by 8~125 fold and 45~75%, respectively. Moreover, SFN is able to reduce ALDH^+^ population in NOD/SCID xenograft tumors after daily injections of 50 mg SFN per kilogram of animal. Cancer cells harvested from mammary xenograft tumors and subsequently treated with SFN when re-implanted in a secondary mouse had their tumor-initiating potential significantly abrogated (54).

### 5.4 Prostate cancer

There is growing evidence that prostate CSCs (PCSCs) are involved in prostate cancer oncogenesis and metastatic process. PCSCs were firstly identified by Collins et al. (55) as CD44^+^α2β1^hi^CD133^+^ prostate cancer cells. More recently, it was found that PCSCs also expressed the breast cancer resistance protein/BCRP ABCG2, some prostate-specific antigens (Trop2^hi^, CD166/ALCAM, PSA^-^/^low^), and ALDH1A1 (56).

Promotion of migration and invasion is associated with the metastasis process, which affects over one-third of prostate cancer patients leading to poor prognosis irrespective of treatment (surgery, chemo and radiotherapy). SFN was reported to inhibit DU145 cell line invasion through ERK1/2 regulation. By promoting ERK1/2 phosphorylation, SFN downregulated protein accumulation of CD44v6 and MMP-2, and increased E-cadherin protein levels. While E-cadherin is an invasion inhibitor, CD44v6 and MMP-2 promote cell invasion, thus facilitating metastasis (57). Furthermore, SFN diminished c-Myc-mediated PCSC traits including high ALDH activity, *CD49f* overexpression and sphere forming capacity (58). Therefore, SFN may prevent or repress the dissemination of prostate cancer cells to surrounding or distant tissues, thus avoiding metastasis.

### 5.5 Colon cancer

Colorectal CSCs (CCSCs) were first reported by 59 as CD133^+^ human colorectal cancer (CRC) primary cells. The authors transplanted CD133^+^ CRC cells into the renal capsule of immunodeficient mice and observed that these cells were able to maintain self-renewal and differentiation capabilities leading to tumor formation. Afterward, other cell surface markers were also found to be overexpressed in CCSCs, such as CD44, CD24, LGR5, and ALDH (60).

SFN decreased cell viability and induced apoptosis in HCT116 and RKO CRC cells, as well as exerted epigenetic alterations in these cells by downregulating *HDAC1* and *hTERT* mRNA expression. *hTERT* is commonly upregulated in cancer and is essential for constitutive cell proliferation and for inducing EMT and stemness characteristics in cancer cells (35, 61).

Moreover, it is known that *TAp63α* overexpression promotes self-renewal capacity and increases CSC markers. *TAp63α* is able to bind *LGR5* promoter and enhance its expression leading to the activation of the Wnt-β-catenin pathway. SFN was able to decrease sphere formation capacity of CRC cells and the expression of CCSC markers including *CD133*, *CD44*, *Nanog*, and *Oct-4*. Remarkably, *TAp63α* expression was downregulated upon SFN treatment, impacting the maintenance of CSC traits (62).

### 5.6 Gastric cancer

The existence of Gastric CSCs (GCSCs) has been also reported and plays a role in gastric cancer initiation and progression (63). Zhu and colleagues (37) showed that GCSCs overexpressed CSC markers including *Oct-4*, *Sox2*, *Klf4*, and *CD44* compared to the human gastric cancer cell line SGC7901. On the other hand, *E-cadherin* expression was lower in GCSCs than in SGC7901 cells. The authors also reported that GCSCs promoted gastric cancer invasion, migration, and angiogenesis, suggesting this sub-population of cells as the fuel for gastric cancer development and maintenance (37).

Similar to lung CSCs, SHH pathway plays an important role in the maintenance of GCSCs. This signaling pathway can promote gastric cancer initiation and progression by inducing cancer cells proliferation (64). SFN was found to inhibit the activation of SHH pathway, tumorsphere formation capacity, and also decreased the expression of CSC markers such as *CD133*, *CD44*, *Oct-4* and *Nanog* in gastric cancer cells. Additionally, SFN suppressed proliferation and induced apoptosis of gastric cancer cells (65), impairing the maintenance of GCSCs and consequently gastric cancer tumorigenesis.

Some chemotherapeutic drugs activate signaling pathways that can promote CSC-like properties. For example, high activation of interleukin-6/IL-6 receptor signal transducer and STAT3 signaling pathway was observed in the gastric cancer cells MGC803 and BGC823 treated with CIS in comparison to untreated cells, triggering a CIS-induced CSC-like enrichment. On the other hand, SFN-treated gastric cancer cells exhibited higher activation of *miR-124*, which directly targets the 3′-untranslated regions (UTR) of *IL-6R* and *STAT3*, thus preventing stemness characteristics (66).

### 5.7 Pancreatic cancer

Pancreatic CSCs (PCSCs), that represent approximately 0.5-1% of pancreatic cancer cells (67), are characterized by the expression of the surface markers CD44^+^/CD24^+^, CD133^+^ and ESA^+^, and their presence is associated with poor prognosis (68). The existence of PCSCs was first reported by 69 in a xenographic mouse model of human pancreatic cancer. The authors injected CD44^+^CD24^+^/ESA^+^ cancer cells in immunocompromised mice and observed a 100-fold increase in tumorigenic potential when compared to those mice injected with nontumorigenic cancer cells (69).

SFN-treated pancreatic cancer cells AsPC1, Capan-1, MIA-PaCa-2, and BxPc-3 exhibited a reduction in their clonogenic potential (70). Additionally, SFN was reported to target PCSCs by impairing NF-κB-induced antiapoptotic signaling, thus increasing apoptosis in this cell population. Mechanistically, SFN prevented the activation of NF-κB transcription factors. These factors play an important role in the regulation of programmed cell death and their activation may promote cell resistance against apoptosis (71).

In addition, PCSCs self-renewal capacity was reported to be affected by SFN treatment. CD133^+^/CD44^+^/CD24^+^/ESA^+^ PCSCs isolated from human primary pancreatic tumor were orthotopically injected into NOD/SCID/IL2R gamma mice. SFN decreased the growth of tumors by 45%, and reduced the expression of SHH pathway components *Smo*, *Gli 1*, and *Gli 2* in mouse xenografts. Also, SFN was able to reduce pluripotency and EMT marker expression, including *Nanog* and *ZEB-1*, respectively. In the same study, the angiogenic markers VEGF and PDGFRa were also inhibited by SFN treatment (72).

These studies support evidence that the self-renewal ability of CSCs can be precluded by SFN treatment in a variety of cancer cells, and SFN can effectively inhibit the tumor-initiating capacity of CSCs in murine models. Moreover, SFN treatment seems to disrupt different CSC-related pathways, negatively impacting the maintenance of CSCs and preventing their dissemination to surrounding or distant tissues. These findings highlight the therapeutic potential of SFN and indicate possible benefits of translating this natural product into the clinical setting.

## 6 Therapeutic approaches

Over the last few years, a variety of *in vitro* and *in vivo* studies have been performed to assess the therapeutic potential of natural products derived from herbs (nutraceuticals) against different types of cancer (73). Notably, some studies have suggested SFN as a potent sensitizer or synergistic agent when combined with different chemotherapeutic drugs currently used in the clinic to treat tumors (18, 74–77) (**Table 1**). Furthermore, the capacity of SFN to target CSCs has indicated this nutraceutical as a promising candidate to be employed in anti-CSC therapeutic approaches.

**Table 1:** Therapeutic approaches using sulforaphane for treating different tumors and its effects.

Therapy	Cancer Type	Affected Cancer Cell Population	Model	Effects	References
**SFN**	Prostate Cancer	CSCs	Men on active surveillance for prostate cancer	Dose-dependent inhibition of oncogenic pathways such as TGF-β, Kras, NFκ-B, and Notch.	78
**SFN**	Pancreatic Cancer	CSCs	PANC-1, MIA PaCa-2, AsPC-1 and Bx PC-3 human cell lines	Reduction of EMT-related genes expression including β-catenin, vimentin, Twist-1, and Zeb-1.	74
**SFN + DOX**	Mammary Adenocarcinoma	Non-CSCs	MATB-III rat mammary gland tumor cell line, Sprague Dawley rats; 4T1 murine cell line, Balb/C mice	Inhibition of tumor growth with lower DOX dosage, reduced cardiotoxicity upon activation of Nrf2; Reduction in tumor volume, increase in cytotoxic CD8^+^T cells, decrease in MDSC population with subsequent immunosuppression.	20, 79
**SFN + Nano-metformin**	Breast Cancer	CSCs	MCF-10, MCF-7, and BT-474 human cell lines	Decrease in Wnt1, β-catenin and CD44 expression; While increased Bax expression and cell death.	80
**SFN + Quercetin**	Pancreatic Cancer	CSCs	PANC-1, MIA PaCa-2, AsPC-1 and Bx PC-3 human cell lines	Higher inhibition of self-renewal capacity of PCSCs.	74
**SFN + CIS/DOX/GEM/5-flurouracil**	Pancreatic Cancer	CSCs	MIA-PaCa2 and DU145 human cell lines Balb/C nude mice	Anti-proliferative effects; Abolishment of tumor initiation capacity.	81
**SFN + Curcumin/dihydrocaffeic acid**	Colon Cancer	Non-CSCs	Caco-2 and HT-29 human cell lines	Combination-dependent cytotoxic effects.	77

SFN, Sulforaphane; DOX, Doxorubicin; CSCs, Cancer Stem Cells; MDSC, Myeloid-Derived Suppressor Cell; EMT, Epithelial-to-Mesenchymal Transition; CIS, Cisplatin; GEM, Gemcitabine.

There are different studies showing the benefits of SFN alone or in combination with other compounds (e.g., nutraceuticals, drugs) for treating a variety of cancer cell lines or animal models. 75 reported that SFN enhanced doxorubicin (DOX) cytotoxic effect in a rat orthotopic breast cancer model, inhibiting tumor growth. Interestingly, DOX concentration required to treat tumors could be decreased when SFN was administered simultaneously. Additionally, SFN exerted cardioprotective effects in these rats by reducing DOX cardiac oxidative stress, evidenced by the inhibition of lipid peroxidation and activation of Nrf2 (75). This is remarkable, since DOX-induced cardiotoxicity and heart damage is a challenge in the current therapeutic scenario (82). In another study, syngeneic mice bearing 4T1 mammary tumors treated concurrently with DOX and SFN, exhibited a significant reduction in tumor volume, increased cytotoxic CD8^+^T cells and decreased proliferation of myeloid-derived suppressor cells (MDSCs), reversing the immunosuppressive microenvironment commonly found in tumors (79).

Furthermore, Keshandehghan and collaborators (80) showed that SFN associated with nano-metformin molecules increased apoptosis in breast cancer cells MCF-7 and BT-474, impacting their survival. The co-treatment also reduced the expression of some CSC-related molecules including *Wnt1*, *β-catenin* and *CD44*, while increased *BAX*, a pro-apoptotic molecule. The authors also found that increased cell death was directly correlated with *BAX* levels but inversely correlated with levels of CSC signaling genes, including *CD44* (80). Moreover, the self-renewal capacity of pancreatic CSCs was significantly reduced when SFN was combined with quercetin, another natural product present in many plants and foods, such as apples, grapes, red wine, and berries. In addition, SFN inhibited the expression of EMT-related genes including *β-catenin*, *vimentin*, *twist-1*, and *ZEB1*, suggesting that SFN alone can prevent the first stages of the metastatic process (74, 83).

Due to the antioxidant properties of SFN, concerns were raised about combining it with cytotoxic drugs including CIS, DOX, gemcitabine (GEM), and 5-flurouracil. In this regard, Kallifatidis and collaborators (70) reported that CSC^high^ pancreatic cells co-treated with SFN and CIS/DOX/GEM/5-flurouracil exhibited lower rates of cell viability and decreased clonogenic potential compared to cells treated individually. More importantly, co-treatment abolished tumor initiation capacity of the cells in nude mice (70). These results suggest that the antioxidant properties of SFN do not impact the cytotoxicity of antineoplastic drugs, but on the contrary, seems to improve it.

The synergistic effect of SFN with the nutraceutical, curcumin and dihydrocaffeic acid, was evidenced in colon cancer. The cytotoxic concentrations to kill 50%, 75%, and 90% of the cells were determined, and different treatment combinations were performed and tested against the colon cancer cell lines HT-29 and Caco-2, as well as the normal colon cell line FHC. The interaction between these three nutraceuticals was combination-dependent, since the cytotoxic effects were correlated with the different combinations used as treatment. Interestingly, combination of SFN with curcumin showed a relatively high antagonistic effect in the cells, while SFN plus dihydrocaffeic acid at 1:1 proportion was cytotoxic to cancer cells in comparison to normal cells. Conversely, when SFN was combined with dihydrocaffeic acid at 1:4 and 9:2 proportions, similar or more cytotoxic effects were observed in normal cells (77).

Despite all the potential benefits of SFN presented so far, it seems that not all cancer patients may benefit from its effects. An *in-silico* analysis predicted the SFN-induced adverse effects in CRC patients. It was found that SFN upregulated genes related to CRC promotion, including *TIMP1*, *AURKA*, and *CEP55*, while it downregulated *CRYAB*, *PLCE1*, and MMP28, which are involved in CRC progression. On the other hand, SFN-mediated regulation of *PPARGC1A, ABHD3, FGL2, NEBL*, and *UGDH* could contribute to its anti-tumor effects (84). Therefore, it is necessary to better investigate what tumors and patients would be the candidates to experience the potential benefits of SFN.

## 7 Clinical applications

SFN is found to be well tolerable by humans with only a few side-effects reported, such as constipation, nausea, and intestinal gas/bloating, which makes this nutraceutical a good candidate for being used in clinical settings (85).

A variety of clinical trials have been conducted aiming to assess SFN effects in some cancers and are reported under the clinicaltrials.gov website. A phase II clinical trial investigated whether SFN could lead to a decline of ≥ 50% in Prostate Specific Antigen (PSA) levels in prostate cancer patients. Twenty participants were treated with 200 μmol/day of SFN-rich extracts for a maximum period of 20 weeks and their PSA levels were measured. Although the results showed that only one patient exhibited a ≥ 50% PSA decline, SFN lengthened the PSA doubling time (PSADT) of patients from 6.1 months during pre-treatment to 9.6 months during treatment. PSADT is an indicator of prostate cancer progression and predicts the number of months taken for PSA to increase two-folds. In addition, the treatment proved to be safe, with no grade 3 adverse effects. Once SFN treatment may exert positive effects on PSADT and is safe for use in patients, further studies, especially using higher doses, are required to clarify the role of SFN as a prognostic and/or therapeutic agent for prostate cancer (86).

Moreover, the ESCAPE clinical trial evaluated whether one year of glucoraphanin-rich broccoli soup consumption would lead to gene expression alterations in prostate cancer. A cohort of 49 participants was enrolled in this trial and the control group received a 300 mL portion of soup made from standard broccoli weekly, while the intervention group received the same portion of soup but made from glucoraphanin-enriched experimental broccoli genotypes. RNA sequencing revealed inhibition of oncogenic signaling pathways including TGF-β, KRAS, NF-κB, and Notch, in a dose-dependent manner in the intervention group (**Table 1**). Even though this trial was not designed to assess clinical progression, an inverse correlation between cruciferous vegetables intake and cancer progression was observed, thus indicating the potential benefits of SFN against prostate cancer (78).

A double-blinded, randomized controlled clinical trial was conducted to investigate the chemopreventive effects of SFN in a cohort of 54 women with abnormal mammograms already scheduled for biopsy. The intervention arm (n=27) received a glucoraphanin supplement providing SFN, while the remaining participants (n=27) received placebo. Selected biomarkers were measured from blood and breast tissue before and after treatment, including HDAC, H3K18ac, H3K9ac, HDAC3, HDAC6, Ki-67, and p21. The study showed that Ki-67 and HDAC3 levels significantly decreased in benign breast tissues, and there was also a reduction in HDAC activity in blood cells. However, no significant pre-to-post-intervention changes was observed in the biomarkers between treatment groups, suggesting that SFN supplementation may be safe, but not sufficient for promoting changes in these tumor markers (87). On the other hand, in a complimentary analysis of the same clinical trial, 88 showed that SFN intake was inversely associated with Ki-67 protein levels in ductal carcinoma in situ, while no changes were observed in benign tumors or invasive ductal carcinomas (88).

Additionally, a clinical trial with 300 participants exposed to airborne pollutants was conducted to assess the ability of SFN in promoting pollution clearance. There was a significant increase in the excretion of acrolein (23%), a glutathione-derived conjugate of benzene, in the intervention arm, suggesting that SFN may exert chemopreventive effects against lung cancer by promoting the clearance of lung cancer-related agents (89). Currently, the phase II clinical trial NCT03232138, ongoing at the University of Pittsburgh, PA, USA, intends to evaluate the chemopreventive effects of SFN against lung cancer in a cohort of 72 former smokers at high risk for developing this type of cancer (90).

More clinical trials supporting the prognostic and therapeutic potential of SFN using effective formulation and administration methods are warranted to better understand its effects and use as a co-adjuvant nutraceutical agent for treating different types of cancer.

## 8 Conclusion

Plant-derived compounds, including ITCs, represent important sources of chemopreventive and anticancer agents. The naturally occurring product SFN can prevent tumor establishment *in vitro* and *in vivo* by modulating a variety of biological processes such as enzymatic detoxification of carcinogens, oxidative stress, cell cycle arrest, apoptosis, and EMT. Importantly, several studies have shown the ability of SFN to target CSCs in different cancer types, highlighting the impact of this nutraceutical in preventing drug resistance, metastasis, and tumor relapse. Furthermore, the combination of SFN with other natural compounds and cytotoxic drugs have shown promising results. Therefore, the chemopreventive, anticancer and anti-CSCs potential of SFN and its use as a co-adjuvant agent deserves further clinical investigation.

## Author contributions

LC was responsible for doing the research and writhing tasks, while MR and TT contributed by editing and making improvements to make the text more cohesive and coherent. All authors contributed to the article and approved the submitted version.
